# Three-Dimensional Thermo–Chemo–Mechanical Coupled Curing Analysis for the Filament Wound Composite Shell

**DOI:** 10.3390/polym16121643

**Published:** 2024-06-10

**Authors:** Linjiao Lu, Shengsheng Huan, Mengkai Lu, Tao Shen, Yanhui Tian, Jianying Hu, Jianke Du, Minghua Zhang

**Affiliations:** Zhejiang-Italy Joint Lab for Smart Materials and Advanced Structures, School of Mechanical Engineering and Mechanics, Ningbo University, Ningbo 315211, China; 2111081018@nbu.edu.cn (L.L.); 2111081104@nbu.edu.cn (S.H.); shentao@nbu.edu.cn (T.S.); 2311090032@nbu.edu.cn (Y.T.); hujianying@nbu.edu.cn (J.H.); dujianke@nbu.edu.cn (J.D.)

**Keywords:** curing kinetics, thermo–chemo–mechanical coupled, composite shells, curing deformation, residual deformation

## Abstract

Carbon fiber resin-based composite materials are widely employed in the manufacturing of composite shells. During the curing process, the temperature gradients and cure degree gradients make it easy to generate thermal strains in both carbon fibers and resin, with the resin experiencing cure shrinkage strain due to the curing reaction, ultimately leading to residual stresses and strains. In this paper, a three-dimensional thermo–chemo–mechanical coupled curing model of the composite shell was established based on a resin test, and the changes of temperature, curing degree, residual stress, and strain during the solidification of the composite shell were investigated. First, the curing property parameters and elastic modulus of HCM-2184 resin were obtained through a curing dynamic test and a tensile test. Then, considering the heat release and shrinkage reaction of solidification, a coupled thermo–chemo–mechanical curing model was developed with the CHILE (α) elastic model, and the curing process of the composite shell was simulated numerically. The results show that the resin used in the test belongs to the autocatalytic reaction. For thin composite shells, the heat accumulation inside the shell during curing is not obvious. During the curing process, the curing shrinkage behavior of the resin is an important factor for the generation of residual stress and residual strain.

## 1. Introduction

Composite materials, which have been widely applied in recent years, typically comprise two or more materials with distinct physical or chemical properties at the macroscopic scale. These materials not only retain the advantages of their constituent components, but also exhibit superior comprehensive performance compared to single materials at the macro level. Compared to traditional metallic materials and alloys, carbon fiber resin-based composites possess advantages such as low density, high-temperature resistance, corrosion resistance, high strength, high modulus, fatigue resistance, and a low thermal expansion coefficient [1,2,3,4], making them extensively used in aerospace and other fields [5]. For instance, at the same volume, ensuring strength and safety while reducing mass can effectively decrease the load. Additionally, in high-temperature and high-humidity environments, the material properties remain substantially unchanged, aligning with the lightweight manufacturing requirements of modern solid rocket engines, hence justifying their use in engine shell fabrication. The resin, serving as the matrix of composite materials, exhibits curing behavior, a chemical change where under external heating, internal groups within the resin undergo cross-linking reactions, releasing heat and transitioning from a viscous flow state to a glassy state [6], with an increase in viscosity and volume shrinkage. During the curing process of composite material shells, the presence of thermal expansion and contraction between carbon fibers and resin, resin cure shrinkage [7,8,9], and mismatches in the thermal expansion coefficients between different parts of the shell and mold materials [10] lead to thermo–chemo–mechanical coupled residual stresses and strains during the curing and molding process. After curing, some residual stresses remain within the shell, causing deformation that deviates from the designed dimensions, affecting the molding quality of shell components and their performance indicators. For example, in the strength prediction of the shell, the residual stresses and strains formed during curing can be considered to be prestress and prestrain. Therefore, the analysis of shell curing, as a factor affecting the performance of molded shells, necessitates the study of the evolution and distribution patterns of residual stresses and strains generated during curing.

The analysis of the structural quality of composite material components during curing molding essentially involves the study of changes in internal stresses and strains within the components caused by the combined effects of carbon fiber and resin thermal expansion, as well as resin curing, under the segmented processes of heating, holding, and cooling during the curing process. Currently, numerous researchers have employed experimental and numerical simulation approaches to research the curing process of composite materials based on thermosetting resins [11,12,13,14,15]. The study of resin cure kinetics forms a crucial foundation for investigating the curing deformation of composite materials. Differential Scanning Calorimetry (DSC) is commonly used to study resin curing behavior. Lee et al. [16] obtained the kinetic parameters of 3501-6 resin by DSC. Zanjanijam [17] studied the curing kinetics of phenolic resin/coal coke composites.

The curing process significantly impacts the quality of curing molding of composite materials. On the one hand, temperature affects the curing progression of the resin, leading to phenomena of uneven curing. On the other hand, the properties of resin [18,19] also change with temperature variations, such as thermal conductivity, density, and specific heat capacity. Pedersen et al. [20] investigated the thermal diffusivity of epoxy resin during the curing process, and discovered that thermal conductivity and specific heat capacity have functional relationships with the degree of cure. Johnston [21] studied the density and various thermophysical parameters of AS4/8552 composite materials.

The simulation of composite materials’ curing has undergone a developmental process [22,23,24]. Cheung [25] integrated thermal conduction with resin cure kinetics to study the temperature field distribution of composite laminate boards during the curing process. Abdelal [26] utilized thermodynamic parameters that change with temperature to construct a thermo–chemical coupled model, calculating the temperature and cure degree distribution of composite material components. To accurately simulate and predict the evolution of stress and strain during the curing process of composite material components, it is essential to establish their constitutive model throughout the entire process, and a substantial number of scholars have researched the constitutive models of composite materials. Among them, the main categories are linear elastic and viscoelastic constitutive models. Bogetti and Gillespie [27] proposed a curing-hardened instantaneous linear elastic model, assuming that the resin behaves linearly elastically at each incremental step during the curing process, and considering that the elastic modulus has a functional relationship with the degree of cure, hence referred to as the CHILE (α) model. However, the above methods did not account for the viscoelasticity of composite materials and the stress relaxation it causes. In order to more accurately characterize the stress relaxation behavior of composite materials, researchers introduced viscoelasticity into the calculations for the curing of composite materials [28,29,30], with Ding et al. [31,32] establishing a thermo–viscoelastic constitutive model considering stress relaxation based on a generalized Maxwell model with n Maxwell elements. The thermo–chemo–mechanical coupled curing process of laminates was numerically simulated. Nonetheless, due to the significant difficulties in measuring the viscoelastic parameters of composite materials and the increased complexity of numerical calculations involving viscoelastic constitutive equations, the CHILE (α) model, by comparison, is relatively simple and can more accurately predict the stress evolution during the curing of composite materials, and is thus widely adopted by scholars.

As aforementioned, the fully coupled analysis for thermo–chemo–mechanical curing is very important. While at present, there are few numerical simulation studies on the application of specific types of resins in the curing of filament wound composite shells, this article addresses the issue of residual stresses and strains leading to structural deformation during the curing process of composite material shells. The test was conducted using HCM-2184 resin. Differential scanning calorimetry (DSC) was used to test the curing kinetics of the resin, and the curing properties of the resin were obtained. A tensile test was carried out to study the tensile properties of resin-casting parts. Based on the consideration of resin-curing heat release, curing shrinkage, and anisotropy of composite materials, a thermo–chemo–mechanical coupled curing model of the filament wound composite shell was established with the CHILE (α) constitutive model. The evolutionary distribution of temperature, curing degree, and residual strain during curing molding was studied, which provided a reference for the quality evaluation of curing molding.

## 2. Resin Test

### 2.1. Preparation of Resin Matrix

Material: HCM-2184 epoxy resin and curing agent, XTEND832 semi-permanent release agent. First, the HCM-2184 epoxy resin was mixed with the curing agent at the mass ratio 100:31. Then, through the high speed stirring and mixing evenly, the resin was vacuumed in the vacuum oven at room temperature for an hour. Finally, when no bubbles appeared, the resin was removed, and subsequent tests were carried out.

### 2.2. Non-Isothermal DSC Test

In order to study the curing reaction kinetics of HCM-2184 epoxy resin, according to the curing characteristics of HCM-2184 epoxy resin, Netzsch DSC-200F3 non-isothermal DSC tests were carried out. The 5 mg mass sample was placed in an aluminum pot, heated to 300 °C at the heating rate of 5.0, 10.0, 15.0, and 20.0 °C/min, and tested under nitrogen atmosphere.

### 2.3. Tensile Test

**Figure 1:**
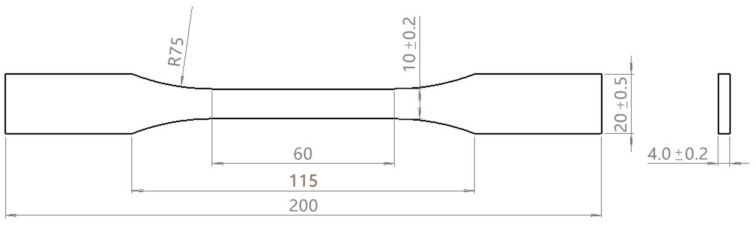
Tensile sample size (unit: mm).

When calculating the curing process of composite materials, it is necessary to input the elastic modulus parameters of the resin. Therefore, tensile tests on the resin are required. The tensile test specimens of the resin are prepared according to the current national standard in China [33], with specific dimensions, as shown in Figure 1.

### 2.4. Experimental Results and Discussions

**Figure 2:**
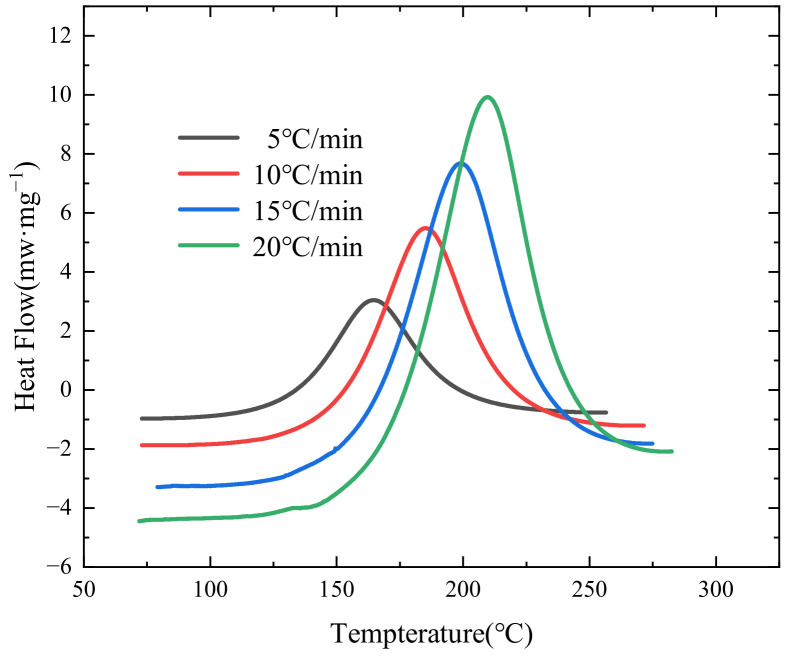
Results of non-isothermal DSC testing of HCM-2184 resin.

The DSC heat flow diagram of epoxy resin at different heating rates (5 °C/min–20 °C/min) is shown in Figure 2. It can be seen that there is a single heat release peak at different heating rates, and the peak heat flow and peak temperature increases with the increase in heating rate, and the heat release peak moves towards the higher temperature direction with the increase in heating rate, indicating that the resin has a heat transfer lag phenomenon.

According to the results of Figure 1, the characteristic parameters such as maximum heat release temperature (peak temperature), peak heat flow, and total heat release of curing reaction ($\Delta H_{cure}$, calculated by the area under the peak heat release) are summarized in Table 1, and the figures in brackets are standard deviations. The results of each heating rate were averaged by repeated tests (at least three times), and the total curing heat release calculated at different heating rates was averaged to obtain the final reaction heat of the resin.

To solve the cure kinetics equation of the resin, it is necessary to determine the resin’s activation energy through mathematical fitting. Activation energy is defined as the minimum energy required for a chemical reaction to occur: the smaller the activation energy, the more readily the reaction takes place. Commonly used equations for calculating activation energy include the Kissinger equation and the Ozawa equation [34]. This article employs both equations to calculate the activation energy of the resin, and the average of the two results is taken as the final activation energy.

The Kissinger equation is expressed as follows:

(1) $$\ln(\frac{\tau }{T_{P}^{2}})=\ln(\frac{AR}{E_{a}})-\frac{E_{a}}{R}\frac{1}{T_{P}},$$

In the equation, R represents the ideal gas constant, generally taken as 8.314 J/(mol·K); A denotes the frequency factor, with the unit $S^{-1}$. $E_{a}$ is the activation energy of the cure reaction being determined, τ is the heating rate, and $T_{p}$ is the peak temperature obtained from tests under different heating rates.

The Ozawa equation is expressed as follows:

(2) $$\frac{d\left(\ln\tau \right)}{d\left(\frac{1}{T_{p}}\right)}=-1.052\frac{E_{a}}{R},$$

In Equation (2), the physical significance of each parameter is the same as that in the Kissinger equation.

**Figure 3:**
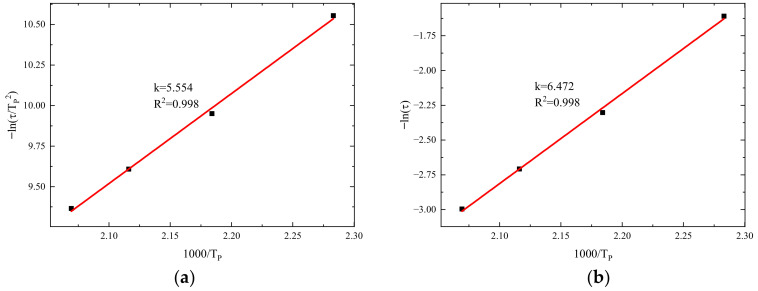
Fitting of activation energy: (**a**) the Kissinger equation; (**b**) the Ozawa equation.

**Figure 4:**
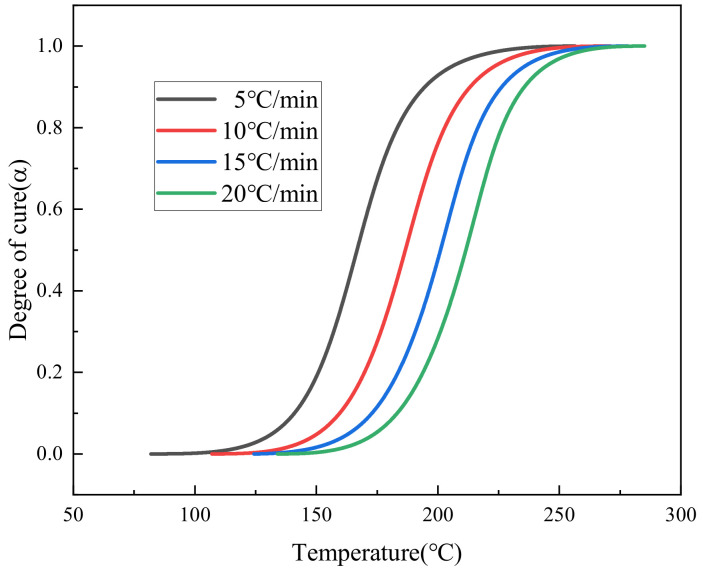
Changes in curing degree of the HCM-2184 resin at different heating rates.

Firstly, the activation energy is determined based on the Kissinger equation. Using the data from Table 1, a plot is constructed with ${1000/T}_{p}$ as the x-axis and $-\ln(\tau /T_{p}^{2})$ as the y-axis. After linear fitting, the result is presented in Figure 3a. Using the Ozawa equation to determine the activation energy, a plot is created with ${1000/T}_{p}$ as the x-axis and −ln⁡(τ) as the y-axis. After linear fitting, the result is shown in Figure 4.

In Figure 3a, after linear fitting of the HCM-2184 resin, the slope of the line is 5.554, with $R^{2}$ = 0.998 indicating a good fit. The reaction activation energy is calculated according to Equation (1), resulting in $E_{a}$ = 46.18 kJ/mol. In Figure 3b, the slope of the line is 6.472, with $R^{2}$= 0.998. The reaction activation energy is calculated according to Equation (2), $E_{a}$ = 51.15 kJ/mol.

Taking the average of the results calculated from the two equations, the activation energy for HCM-2184 resin is determined to be 48.67 kJ/mol.

When calculating the degree of cure (α) during the curing process of thermosetting resin, it can be represented by the ratio of the heat of reaction at the current time to the total heat of reaction, as shown in Equation (3). Among them, the reaction heat at the current moment ($\Delta H_{T}$) is obtained by integrating the heat flow curve measured using DSC in Figure 1 over time, and the total heat released during the curing process is shown in Table 1:

(3) $$\alpha =\frac{\Delta H_{T}}{\Delta H_{cure}},$$

The variation trend of the curing degree of HCM-2184 resin with the increase in temperature under different heating rates is obtained, as shown in Figure 4.

**Figure 5:**
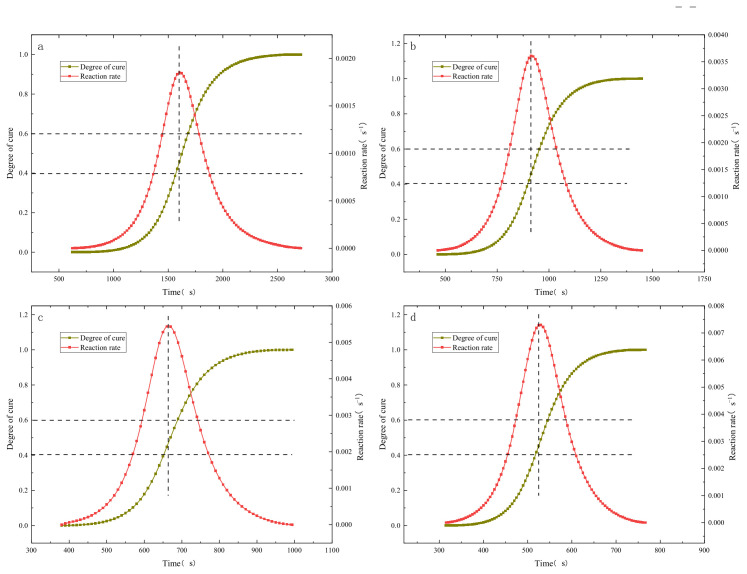
Comparison of curing degree and curing rate at different heating rates: (**a**) 5 °C/min; (**b**) 10 °C/min; (**c**) 15 °C/min; (**d**) 20 °C/min.

By further deriving the degree of cure with respect to time, the relationship between the cure reaction rate of the resin and time at different heating rates is obtained. Curves depicting the degree of cure and cure rate as functions of time under four different heating rates are plotted, as shown in Figure 5. In the figure, the times at which the cure rate reaches its peak are marked vertically, and the corresponding degree of cure intervals are marked horizontally. It can be observed that, at different heating rates, the resin reaches its maximum cure reaction rate within a degree of cure range of 0.4–0.6, indicating that the cure reaction is most intense within this interval.

The N-order reaction model [35] is applicable to the resin system without autocatalytic behavior in the curing reaction, and its expression is shown in Equation (4):

(4) $$\frac{d\alpha }{dt}=K(T)(1-\alpha {)}^{n},$$

where *n* is the order of reaction associated with the material, K is a function of temperature, and α is the degree of cure.

The curing kinetic model expression of epoxy resin system with autocatalytic characteristics is shown in Equation (5):

(5) $$\frac{d\alpha }{dt}=K(T)\alpha^{m}(1-\alpha {)}^{n},$$

**Figure 6:**
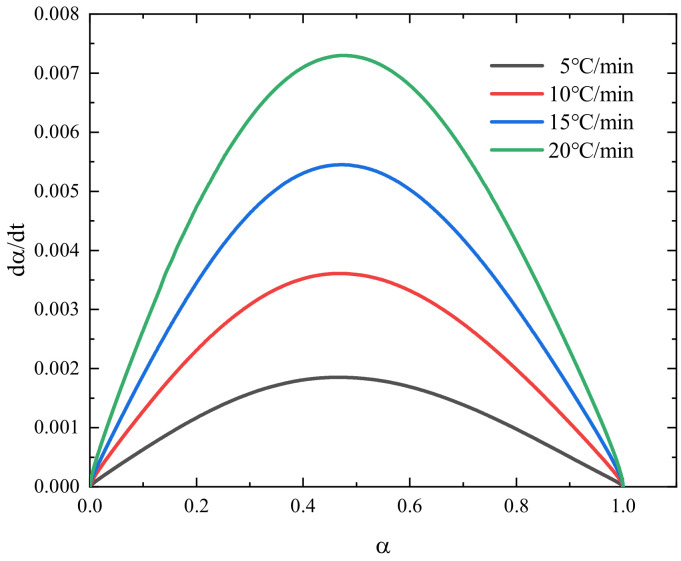
Relationship between the curing reaction rate and curing degree of the HCM-2184 resin at different heating rates.

The relationship between the cure reaction rate and the degree of cure for epoxy resin at different heating rates is shown in Figure 6.

As shown in Figure 6, the cure reaction rate is related to the heating rate, with the cure rate significantly accelerating as the heating rate increases. At four different heating rates, the cure reaction rate exhibits a parabolic trend of first increasing and then decreasing, rather than reaching the maximum value at the outset. The interval at which the cure reaction rate peaks is within the range of 0.4–0.6, largely unaffected by the heating rate. This indicates that the basic model of the resin’s cure reaction is independent of the heating rate, thereby suggesting that the cure kinetics model for the HCM-2184 resin system belongs to an autocatalytic reaction [35].

**Figure 7:**
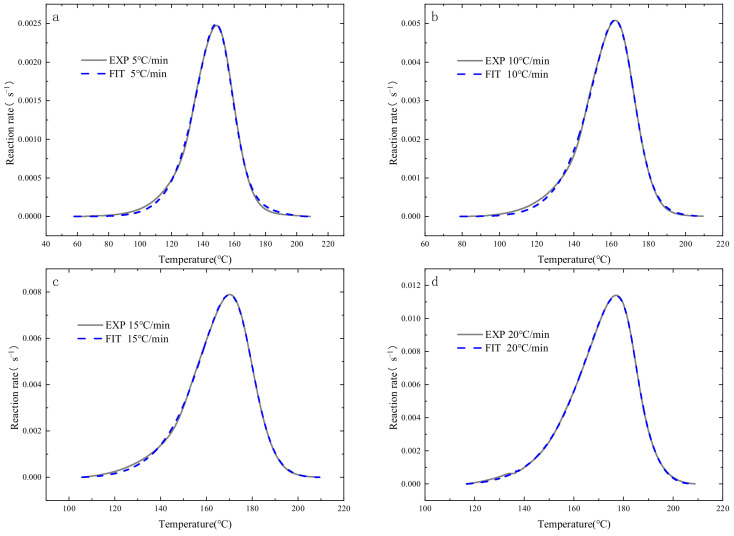
Curve fitting results of the curing rate with different heating rate: (**a**) 5 °C/min; (**b**) 10 °C/min; (**c**) 15 °C/min; (**d**) 20 °C/min.

The cure rate curves obtained from the experiments were subjected to nonlinear fitting based on the autocatalytic reaction model. Studies indicate that the extremum of the resin’s autocatalytic reaction generally occurs at a degree of cure around 0.4. Therefore, to fit the curves more accurately, a degree of cure of 0.4 was used as a demarcation point for segmental fitting. Figure 7 presents a comparison between the experimental and fitting results of the cure rate of HCM-2184 resin at different heating rates. It is observable that, at all four heating rates, the experimental and fitted curves are in basic agreement, indicating that fitting each curve individually yields good results. Subsequently, by averaging the characteristic parameters of the cure kinetics equations fitted at different heating rates, a universal formula is obtained as follows:

(6) $$\frac{d\alpha }{dt}=\left\{\begin{array}{c} 3802*\exp(-\frac{48670}{8.314*T})\alpha^{0.539}(1-\alpha {)}^{1.002},\alpha \leq 0.4\\ 3891*\exp(-\frac{48670}{8.314*T})\alpha^{0.404}(1-\alpha {)}^{1.264},\alpha >0.4 \end{array}\right.,$$

**Figure 8:**
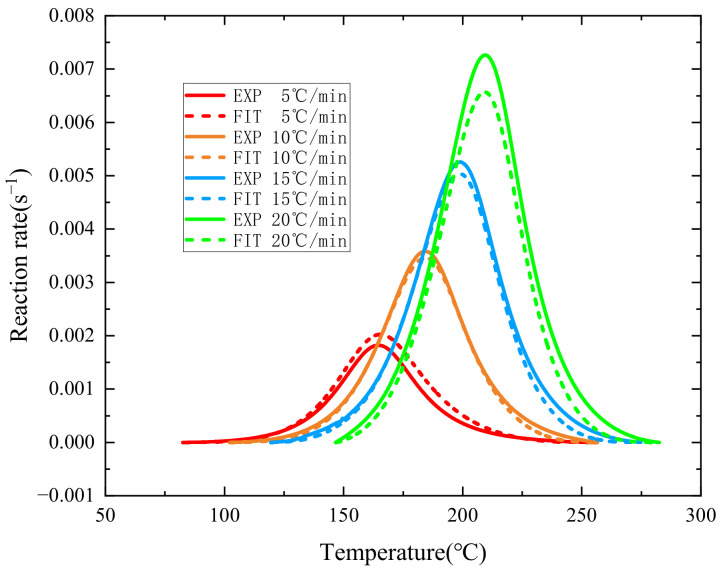
Average result of the curing rate fitting curve.

Figure 8 presents the comparison results of the curves, revealing that, although there is a certain discrepancy between the averaged curves and the experimental curves, they still essentially coincide. At the relatively lower heating rates of 5 °C/min and 10 °C/min, the fitted curves and experimental curves almost overlap. At the higher heating rates of 15 °C/min and 20 °C/min, there is a noticeable difference in the maximum values of the cure rates of the fitted curves, and yet the general trend of the cure rate remains fundamentally the same.

**Figure 9:**
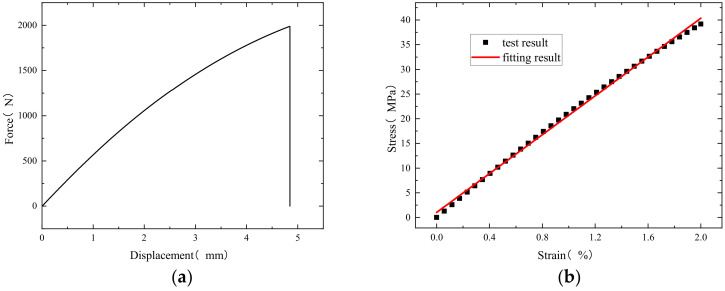
(**a**) Force-displacement curve; (**b**) stress–strain curve and fitting results.

For the tensile test, the force-displacement curve measured using the test is shown in Figure 9a, and the stress–strain curve is drawn with the linear section. The result after linear fitting is shown in Figure 9b. The slope—that is, the elastic modulus of the resin after curing—is 1.97 GPa, which is used as the material parameter for the subsequent curing simulation.

## 3. Construction of the Composite Curing Coupled Model

### 3.1. The Thermo–Chemo–Mechanical Coupled Model

The curing process of resin-based carbon fiber composite material is essentially the result of the joint action of the temperature applied externally and the internal heat source generated from the resin releasing heat. The external applied temperature belongs to the heat conduction, and the curing of the resin belongs to the chemical reaction. The heat conduction and curing reaction in composites lead to the generation of residual stress and strain, resulting in curing deformation. Therefore, the curing process is a thermo–chemo–mechanical coupled process. The three-dimensional Fourier heat conduction equation of the anisotropic composite and the internal heat source of the resin are shown as follows:

(7) $$\rho_{c}C_{p}\frac{\partial T}{\partial t}=k_{11}\frac{\partial^{2}T}{\partial x^{2}}+k_{22}\frac{\partial^{2}T}{\partial y^{2}}+k_{33}\frac{\partial^{2}T}{\partial z^{2}}+{\dot{q}}_{0},$$

(8) $${\dot{q}}_{0}=\rho_{r}V_{r}H_{r}\frac{d\alpha }{dt},$$

where $\rho_{c}$ is the density of the composite material, $k_{ii}$ denotes the heat conduction coefficient of the composite material, $C_{p}$ is the specific heat capacity, ${\dot{q}}_{0}$ is the internal heat source generated during the curing process of the resin, $\rho_{r}$ is the density of the resin, $V_{r}$ is the volume fraction of the resin, $H_{r}$ is the total heat released after the resin is completely cured by the test, and dα/dt is the curing rate.

For the resin used in this paper, a semi-empirical phenomenological kinetic model based on macro-scale was adopted. According to the test, HCM-2184 was determined to be an autocatalytic resin, and the curing rate equation is shown as Equation (6).

The mechanical behavior during the curing process of thermo–chemo–mechanical coupled, that is, the constitutive relationship of the composite material, is described in Section 3.2.

### 3.2. Constitutive Relationships of the Composite Material 

In carbon fiber-reinforced resin composite materials, the carbon fibers acting as the reinforcement material only undergo thermo–mechanical changes during the curing process, and their constitutive behavior is linear elastic. In contrast, the resin, serving as the matrix, is a polymer material that undergoes chemical changes [36] during curing, and generally exhibits viscoelastic behavior. Scholars have proposed computational models for the evolution of modulus during the resin’s curing process, such as the CHILE model, the path-dependent model, and the viscoelastic generalized Maxwell model, among which the CHILE model is widely adopted. It assumes that the resin can be approximately considered to be a linear elastic material within a single time step, with the model linearly varying with the degree of cure, as shown in Equation (9).

(9) $$E_{r}=\left\{\begin{array}{l} E_{r}^{0}\\ (1-\alpha_{mod})E_{r}^{0}+\alpha_{mod}E_{r}^{\infty }\\ E_{r}^{\infty } \end{array}\;\;\right.\begin{array}{l} \alpha <\alpha_{c1}\\ \alpha_{c1}\leq \alpha <\alpha_{c2}\\ \alpha \geq \alpha_{c2} \end{array},$$

where $\alpha_{mod}=(\alpha -\alpha_{gel})/(1-\alpha_{gel})$, $\alpha_{gel}$ is the gel point when the resin starts crosslinking. $E_{r}^{0}$ and $E_{r}^{\infty }$ are the elastic moduli of the resin in the uncured state and when curing is completed, respectively. $E_{r}^{\infty }/E_{r}^{0}$ is usually set to be one thousand. In this case, $E_{r}^{\infty }$ = 1.97 GPa, $E_{r}^{0}$ = 1.97 MPa. $\alpha_{c1}$, and $\alpha_{c2}$ are the curing degrees corresponding to the starting and ending points of curing shrinkage, respectively.

**Figure 10:**
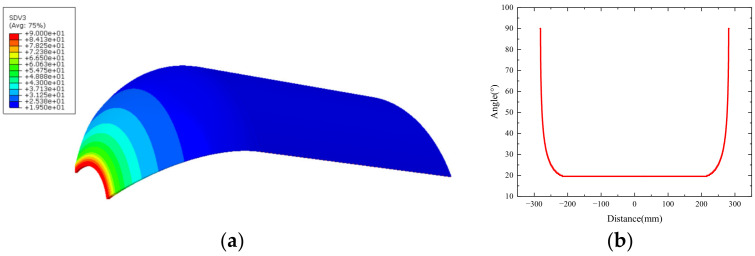
(**a**) Winding angle distribution of the spiral layer; (**b**) the winding angle of the spiral layer changes along the shell axial direction.

The composite shell is composed of fiber winding, which is mainly divided into a barrel body, a pole hole, and a head. Fiber winding is divided into two types: spiral winding, and circumferential winding. The circular winding is only distributed in the body, and the angle is about 90°. In the composite shell model with spiral fiber winding, the winding angle from the barrel to the pole hole is calculated for the fiber winding layer as follows:

(10) $$\alpha (r)=\sin^{-1}(\frac{r_{0}}{r}),$$

where $r_{0}$ is the radius at the polar hole of the shell, and r is the radius of the fiber winding layer at any position of the head section. When r =$\;r_{0}$, the winding angle is 90°, that is, the fiber winding layer is tangent to it at the polar hole. The initial winding angle of the spiral winding layer in the barrel body is 19.5°. When approaching the pole hole, the winding angle increases to 90°. The circumferential winding layer is only distributed in the barrel body, and the angle is 90°, as shown in Figure 10a. Figure 10b shows the change of the winding angle of the spiral winding layer along the axial direction of the shell.

In the actual winding process, the direction of the fibers continuously changes with the winding angle. Therefore, it is unreasonable to place the direction of the fiber material in the global coordinate system, which requires two coordinate rotations.

**Figure 11:**
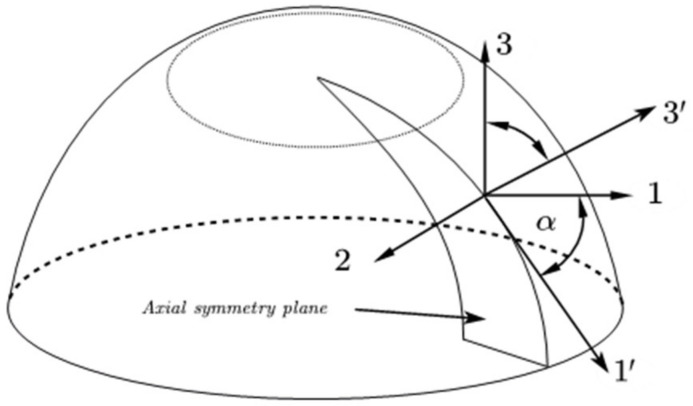
Schematic of the first rotation.

During the first rotation, the entire shell is placed in a cylindrical coordinate system, with the 1, 2, and 3 axes representing the radial, circumferential, and axial directions, respectively. For the end-cap section shown in Figure 11, take the 2-axis as the rotation axis, rotate the 1–3 plane by an angle α, and the rotated plane is a 1′–3′ plane. α is the angle between the original radial axis 1 and the axis 1′ along the generatrix direction of the frustum of a cone.

**Figure 12:**
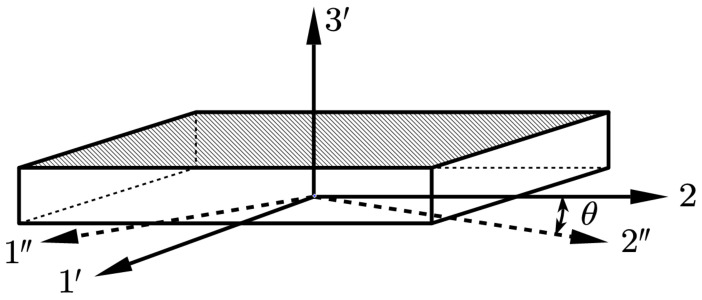
Schematic of the second rotation.

The second rotation is a plane rotation, taking the 3′ axis as the rotation axis, rotating from the 1′–2 plane to the 1″–2″ plane, and the rotation angle θ is the fiber winding angle α(r) calculated by Equation (10). The rotation process is shown in Figure 12.

The winding shell is composed of composite materials, which are considered transversely isotropic materials. The material stiffness matrix is denoted as $\left[C\right]$, where each parameter represents the composite material properties calculated using micromechanical equations for composite materials, incorporating both carbon fibers and resin.

After the rotation, the constitutive equation incorporating angular variables is formed, with the stress–strain relationship as given in Equation (11). $\left[C\prime \right]$ represents the stiffness matrix of the composite material after coordinate transformation.

(11) $$\left\{\begin{array}{c} \sigma_{x}\\ \sigma_{y}\\ \sigma_{z}\\ \tau_{xy}\\ \tau_{xz}\\ \tau_{yz} \end{array}\right\}={\left[T_{\sigma }\right]}^{-1}\left[C\right]\left[T_{\varepsilon }\right]\left\{\begin{array}{c} \varepsilon_{x}\\ \varepsilon_{y}\\ \varepsilon_{z}\\ \gamma_{xy}\\ \gamma_{xz}\\ \gamma_{yz} \end{array}\right\}=\left[C^{\prime }\right]\left\{\begin{array}{c} \varepsilon_{x}\\ \varepsilon_{y}\\ \varepsilon_{z}\\ \gamma_{xy}\\ \gamma_{xz}\\ \gamma_{yz} \end{array}\right\},$$

The stress and strain transfer matrices are given as follows:

(12) $$\left[T_{\sigma }\right]=\left[\begin{array}{cccccc} \cos^{2}\theta & \sin^{2}\theta & 2\sin\theta \cos\theta & 0 & 0 & 0\\ \sin^{2}\theta & \cos^{2}\theta & 0 & -2\sin\theta \cos\theta & 0 & 0\\ 0 & 0 & 1 & 0 & 0 & 0\\ -\sin\theta \cos\theta & \sin\theta \cos\theta & 0 & \cos^{2}\theta -\sin^{2}\theta & 0 & 0\\ 0 & 0 & 0 & 0 & \cos\theta & -\sin\theta \\ 0 & 0 & 0 & 0 & \sin\theta & \cos\theta \end{array}\right],$$

(13) $$\left[T_{\varepsilon }\right]=\left[\begin{array}{cccccc} \cos^{2}\theta & \sin^{2}\theta & 0 & \sin\theta \cos\theta & 0 & 0\\ \sin^{2}\theta & \cos^{2}\theta & 0 & -\sin\theta \cos\theta & 0 & 0\\ 0 & 0 & 1 & 0 & 0 & 0\\ -2\sin\theta \cos\theta & 2\sin\theta \cos\theta & 0 & \cos^{2}\theta -\sin^{2}\theta & 0 & 0\\ 0 & 0 & 0 & 0 & \cos\theta & -\sin\theta \\ 0 & 0 & 0 & 0 & \sin\theta & \cos\theta \end{array}\right],$$

On the other hand, the equation of the linear elastic constitutive equation is as follows:

(14) $$\sigma_{ij}=C_{ijkl}\varepsilon_{kl},$$

(15) $$\left[C\right]=\left[\begin{array}{cccccc} \frac{1-\nu_{23}\nu_{32}}{E_{2}E_{3}\Delta } & \frac{\nu_{12}+\nu_{13}\nu_{32}}{E_{2}E_{3}\Delta } & \frac{\nu_{13}+\nu_{12}\nu_{23}}{E_{2}E_{3}\Delta } & 0 & 0 & 0\\ \frac{\nu_{12}+\nu_{13}\nu_{32}}{E_{2}E_{3}\Delta } & \frac{1-\nu_{13}\nu_{31}}{E_{1}E_{3}\Delta } & \frac{\nu_{23}+\nu_{21}\nu_{13}}{E_{1}E_{3}\Delta } & 0 & 0 & 0\\ \frac{\nu_{13}+\nu_{12}\nu_{23}}{E_{2}E_{3}\Delta } & \frac{\nu_{23}+\nu_{21}\nu_{13}}{E_{1}E_{3}\Delta } & \frac{1-\nu_{12}\nu_{21}}{E_{1}E_{2}\Delta } & 0 & 0 & 0\\ 0 & 0 & 0 & G_{12} & 0 & 0\\ 0 & 0 & 0 & 0 & G_{13} & 0\\ 0 & 0 & 0 & 0 & 0 & G_{23} \end{array}\right],$$

where

(16) $$\Delta =\frac{1-\nu_{12}\nu_{21}-\nu_{23}\nu_{32}-\nu_{13}\nu_{31}+2\nu_{21}\nu_{32}\nu_{13}}{E_{1}E_{2}E_{3}},$$

After coordinate conversion, the constitutive equation including temperature and cure variables can be written as:

(17) $$\sigma_{ij}(t)=\int_{-\infty }^{t}C_{ijkl}(\alpha ,T,t-\tau )\frac{\partial }{\partial t}\left[\varepsilon_{kl}(\tau )-\varepsilon_{kl}^{tc}(\tau )\right]d\tau (i,j,k,l=1,2,3),$$

### 3.3. Flowchart of the Curing Coupled Model

**Figure 13:**
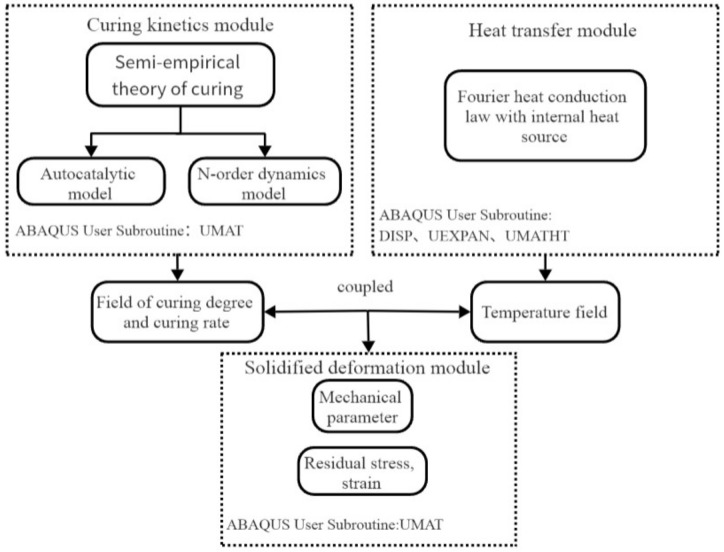
Thermo–chemo–mechanical coupled curing flow chart.

The curing analysis flow chart of thermo–chemo–mechanical coupled composite materials is shown in Figure 13, which is divided into curing dynamics module, heat conduction module and curing deformation module. Among them, the curing dynamics module and heat conduction module are coupled with each other, and the two modules are sequentially coupled with the curing deformation module. The curing calculation of thermo–chemo–mechanical coupling requires the writing of ABAQUS user subroutines. Different modules require different subroutines. The curing kinetics module uses UMAT definitions, the heat transfer module uses DISP, UEXPAN, and UMATHT definitions, and the curing deformation module uses UMAT definitions.

### 3.4. FE Model of the Composite Shell

**Figure 14:**
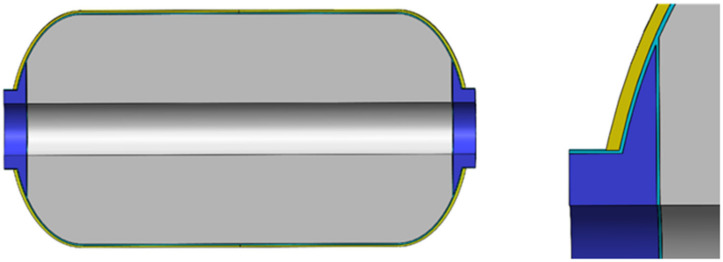
The model diagram of the filament wound composite shell.

A thermo–chemo–mechanical coupled analysis is performed on the whole structure of the carbon fiber composite material shell. The shell consists of carbon fiber winding layers, metal fittings, rubber insulation layers and a sand core mold, as illustrated in Figure 14. In the figure, the blue part represents the metal fittings, the cyan part denotes the rubber insulation layer, the gray part is the sand core mold, and the yellow part is the composite material layer.

**Figure 15:**
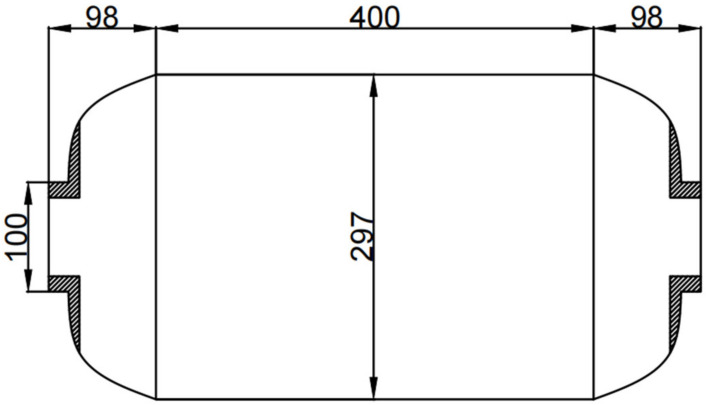
Specific dimensions of composite gas cylinders.

The specific size of the filament wound composite shell is shown in Figure 15. The two ends of the shell have symmetrical polar holes, and the size is the same, the thickness of the rubber insulation layer is 2 mm, the barrel body is 146.5 mm, the length is 400 mm, the distance from the head to the polar hole is 98 mm, the radius of the metal polar hole is 50 mm, and the thickness of the single layer of the fiber winding layer is 0.2 mm. The model was established in the finite element software ABAQUS 2017, and the fiber winding method was [±19.5°/90° ± 19.5°]. The coordinate conversion of material parameters and the calculation of the curing process were carried out by the user subroutine written by ABAQUS.

Apart from resin and carbon fibers, the model incorporates three other materials: metal fittings, the sand core mold, and the rubber insulation layer. The insulation layer is made of ethylene propylene diene monomer (EPDM) rubber, and the front and rear metal fittings are made of 30CrMnSiA material. The mechanical properties of each material are presented in Table 2, while the elastic mechanical parameters of T800 carbon fibers are shown in Table 3.

**Figure 16:**
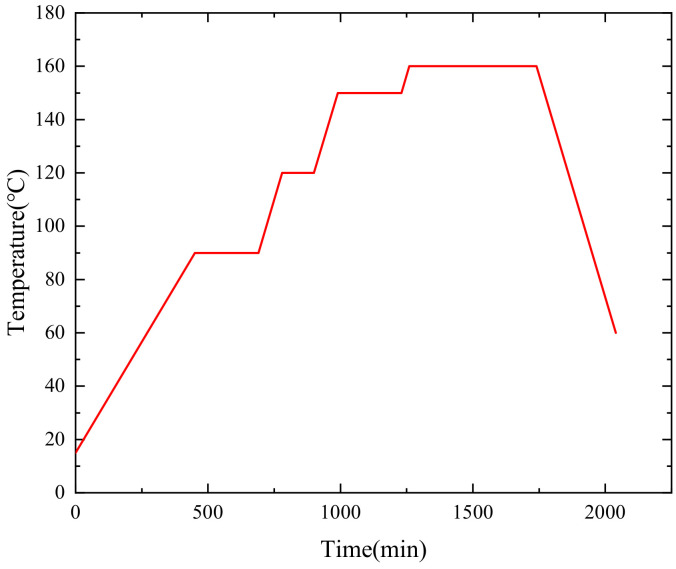
Curing process curve.

The curing process of the composite shell is shown in Figure 16, which is applied to the outer surface of the shell model. The contact type between the fiber winding layer and the insulation layer is the Tie constraint. Considering the symmetry properties of the shell model [37], the 1/36 model is adopted for calculation. The symmetric boundary conditions along the column coordinates are set on both sides of the cross-section to prevent rigid body displacement.

## 4. Results and Discussions

### 4.1. Temperature and Curing Degree Analysis 

**Figure 17:**
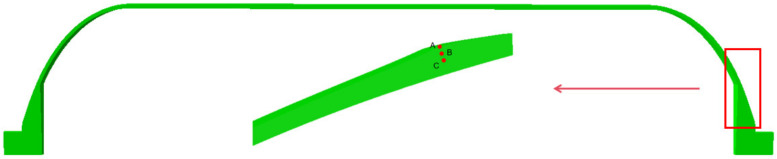
The position of three points A, B, and C of the shell head segment.

A, B, and C are selected as the observation points, which are distributed from the outside to the inside near the polar hole, as shown in Figure 17. 

**Figure 18:**
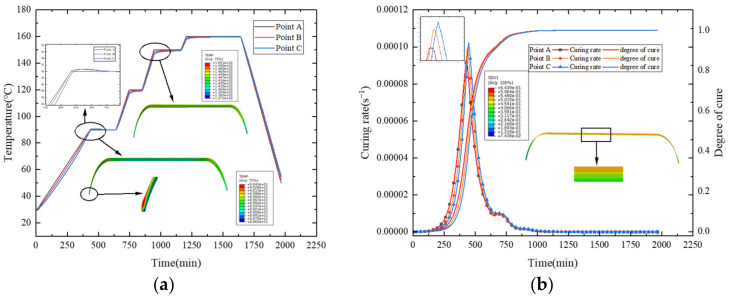
(**a**) Curve of temperature change; (**b**) curve of curing degree and curing rate.

Figure 18a presents the temperature comparison, and Figure 18b compares the degree of cure and cure rate. In the first heating stage, the outer surface is heated, and temperature conducts from the outside inwards, resulting in internal temperatures being lower than external ones. The increase in temperature initiates resin curing, with different temperatures leading to a gradient distribution in the degree of cure, and external curing reactions occurring faster than internal ones. When the heating of the first stage is nearly complete and enters the subsequent heat preservation stage, the temperature is conducted to the interior. The resin begins to undergo intense curing reactions, with the internal degree of cure increasing. During this process, a significant amount of heat is released. In Figure 18a, it can be seen that points B and C have significantly higher temperatures than point A, which is similar to the results in the literature [38]. In addition, it is evident that the concentration of heat causes the fastest cure rate at point C, as observed in Figure 18b. However, due to the thin fiber winding layer of the shell, the phenomenon of heat concentration is not obvious. In the later stages of heating and holding—that is, after about 650 min—no significant accumulation of heat occurs as the resin cure reaction weakens. Hence, the temperature lags behind the curing process. During the cooling stage, internal temperatures are higher than external, conducting heat from the inside outwards, with temperatures gradually approaching the cure temperature.

### 4.2. Residual Stress and Residual Strain Analysis

**Figure 19:**
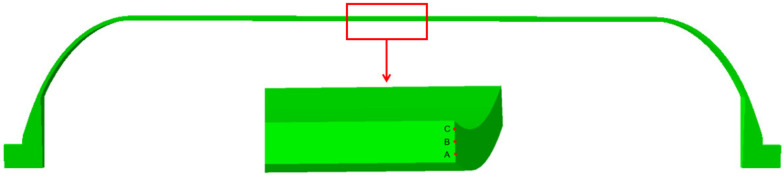
The position of three points A, B, and C of the shell cylinder body.

**Figure 20:**
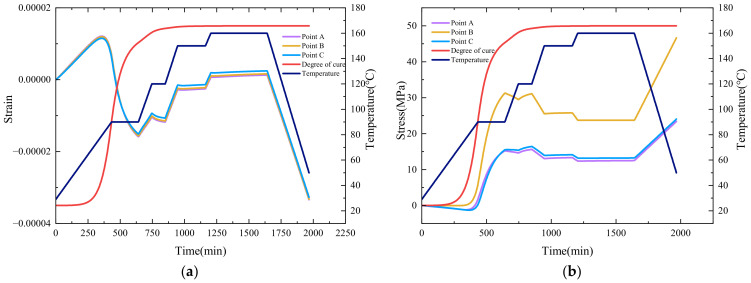
(**a**) Axial stress changes during curing. (**b**) Axial strain changes during curing.

During the curing process, points A, B, and C distributed from the outside to the inside of the cylindrical section of the shell, as shown in Figure 19, are selected to plot the trends of axial residual stress and strain, as illustrated in Figure 20a,b. It can be found that, in the first heating stage, the strain primarily increases due to thermal expansion. At the holding stage, as the resin curing progresses, the strain decreases due to the cure shrinkage effect. When the resin curing is near completion, the strain change is positively correlated with the temperature change, which is similar to the strain change in the literature [39]. When comparing the strain at internal and external points, due to the shell being thin, their strains are essentially the same. 

For the stress situation, the overall trend of stress during the curing process increases. In each heating stage, stress decreases when the temperature rises. In the holding stage before the end of curing, the cure shrinkage of the resin causes a significant increase in stress, indicating that cure shrinkage is a significant factor in the occurrence of residual stress. Comparing the stress at internal and external points, since the strain is similar, and points A and C are in the helical winding layer with the same winding angle, the stress changes are essentially the same. However, point B is in the circumferential winding layer, and after coordinate transformation, the constitutive equation differs from the other two points, resulting in stress being greater at point B than at points A and C.

## 5. Conclusions

In this paper, the results of the curing deformation of filament wound composite shells are studied using experimental and numerical studies. A thermo–chemo–mechanical coupled model for the curing analysis of the composite shell is proposed, which can be used to simulate the curing process and evaluate the quality of the composite shell. The detailed conclusions are listed as follows:

1. The non-isothermal DSC test was carried out on HCM-2184 resin, and the curing reaction was analyzed to belong to the autocatalytic reaction model. The parameters of the curing kinetic formula were obtained by mathematical fitting. The elastic modulus of the resin after curing was obtained using a tensile test. These material parameters are used in the curing numerical simulation.
2. Combining the heat conduction, the curing behavior of resin, and the curing deformation of composite materials, a thermo–chemo–mechanical coupled numerical model was constructed on the basis of considering the coordinate transformation of the composite shell, and the curing process of the composite shell model was simulated.
3. The curing process of the composite shell is analyzed. The results show that the heat accumulation caused by curing heat release can be observed in the thicker parts of the shell, such as the head section. However, due to the thin shell, the curing heat generation is not obvious. During the curing process, the strain changes inside and outside the shell barrel body are close, and the stress is different due to the change in fiber winding angle. During the intensive curing stage of the resin, the strain decreases rapidly and the stress increases, indicating that the curing shrinkage behavior of the resin is an important factor in the generation of residual stress and residual strain.

## Figures and Tables

**Table 1:** Characteristic parameters of resin non-isothermal DSC test.

Heating Rate (°C/min)	Peak Temperature (°C)	Maximum Heat (W/g)	Total Heat Release (J/g)
5	164.85 (0.212)	2.891 (0.214)	1962.50 (60.104)
10	184.70 (0.566)	5.604 (0.173)	1965.00 (74.953)
15	199.40 (0.849)	8.191 (0.714)	1938.00 (82.024)
20	204.75 (0.424)	9.263 (0.651)	1763.00 (67.882)

**Table 2:** Mechanical property parameters of shell material.

Parameter	30CrMnSiA Steel	Core Mold	EPDM Rubber	Unit
E	2 × 10^5^	1 × 10^6^	7.8	MPa
ν	0.33	0.1	0.47	-
k	29.3	0.16	0.26	w·m^−1^·K^−1^
α	1.172 × 10^−5^	1 × 10^−7^	1 × 10^−5^	K^−1^
$C_{P}$	520	1000	2200	J·kg^−1^·K^−1^

**Table 3:** Mechanics property of T800 carbon fiber.

Parameter	Unit	Value
$E_{1}$	MPa	294,000
$E_{2},E_{3}$	MPa	14,000
$G_{12},G_{13}$	MPa	15,000
$G_{23}$	MPa	5500
$\nu_{12}=\nu_{13}$	-	0.23
$\nu_{23}$	-	0.23

## Data Availability

The raw/processed data required to reproduce these findings cannot be shared at this time as the data also form part of an ongoing study.
